# Mechanochemical properties of human myosin-1C are modulated by isoform-specific differences in the N-terminal extension

**DOI:** 10.1074/jbc.RA120.015187

**Published:** 2020-12-03

**Authors:** Sven Giese, Theresia Reindl, Patrick Y.A. Reinke, Lilach Zattelman, Roman Fedorov, Arnon Henn, Manuel H. Taft, Dietmar J. Manstein

**Affiliations:** 1 Institute for Biophysical Chemistry, Fritz-Hartmann-Centre for Medical Research, Hannover Medical School, Hannover, Germany; 2 Division for Structural Biochemistry, Hannover Medical School, Hannover, Germany; 3 Faculty of Biology, Technion-Israel Institute of Technology, Haifa, Israel; 4 Russell Berrie Nanotechnology Institute, Technion-Israel Institute of Technology, Haifa, Israel

**Keywords:** actin, ATPase, myosin, isoform, IQ domain, enzyme kinetics, mechanotransduction, molecular motor, AM, actomyosin, acto·Myo1C, complex of myosin-1C with filamentous actin, A.U., arbitrary units, *K*_app.actin_, apparent dissociation equilibrium constant for actin binding in the presence of ATP, *k*_cat_, maximum value of the steady-state ATPase activity, MDCC-PBP, N-[2-(1-maleimidyl)ethyl]-7-(diethylamino)-coumarin-3-carboxamide-labelled phosphate-binding protein, Myo1C-FL, full-length myosin-1C construct, Myo1C-ΔTH1, myosin-1C construct lacking the TH1 domain, NTE, N-terminal extension, NTE^16^, peptide corresponding to the N-terminal extension of myosin-1 C^16^, NTE^35^, peptide corresponding to the N-terminal extension of myosin-1 C^35^, NTR, N-terminal region, PDB, Protein Data Bank

## Abstract

Myosin-1C is a single-headed, short-tailed member of the myosin class I subfamily that supports a variety of actin-based functions in the cytosol and nucleus. In vertebrates, alternative splicing of the MYO1C gene leads to the production of three isoforms, myosin-1C^0^, myosin-1C^16^, and myosin-1C^35^, that carry N-terminal extensions of different lengths. However, it is not clear how these extensions affect the chemomechanical coupling of human myosin-1C isoforms. Here, we report on the motor activity of the different myosin-1C isoforms measuring the unloaded velocities of constructs lacking the C-terminal lipid-binding domain on nitrocellulose-coated glass surfaces and full-length constructs on reconstituted, supported lipid bilayers. The higher yields of purified proteins obtained with constructs lacking the lipid-binding domain allowed a detailed characterization of the individual kinetic steps of human myosin-1C isoforms in their productive interaction with nucleotides and filamentous actin. Isoform-specific differences include 18-fold changes in the maximum power output per myosin-1C motor and 4-fold changes in the velocity and the resistive force at which maximum power output occurs. Our results support a model in which the isoform-specific N-terminal extensions affect chemomechanical coupling by combined steric and allosteric effects, thereby reducing both the length of the working stroke and the rate of ADP release in the absence of external loads by a factor of 2 for myosin-1C^35^. As the large change in maximum power output shows, the functional differences between the isoforms are further amplified by the presence of external loads.

Myosin-1C connects cell and vesicle membranes with actin-rich structures of the cytoskeleton to support critical cellular processes at multiple intracellular locations. Myosin-1C has been shown to contribute to the adaptation response in sensory hair cells (1), to act as a cofactor of the transcriptional machinery by interacting with RNA polymerase I and II in the nucleus (2, 3), to support the delivery of organelles to membranes such as the insulin-induced translocation of GLUT4-containing vesicles to plasma membrane (4), and to play a role in the formation of membrane extensions and the regulation of cellular tension (5, 6). All myosins share a generic myosin motor domain, which contains an active site and an actin-binding region. Members of different myosin classes have evolved structural modifications to adapt kinetic and mechanical properties to generate force and motion according to their physiological function (7, 8) Myosin-1C is a member of the short-tailed class I myosin subfamily (9). Its generic motor domain is followed by a neck region that serves as a lever arm and consists of three IQ motifs and a post-IQ domain (Fig. 1*A*). IQ1 and IQ2 each bind one calmodulin, while a third calmodulin is bound to both IQ3 and the post-IQ domain (10). The C-terminal 176 residues form the rigid globular tail homology region 1 (TH1), which is found in all members of the class I subfamily. The TH1 domain contains a generic 56-residue, lipid membrane–binding pleckstrin homology (PH) domain in its center. In humans, alternative splicing of the *MYO1C* gene leads to the production of three isoforms, which differ in the length of their N-terminal extension (NTE) (3, 11). Compared to myosin-1C^0^, the isoforms myosin-1C^16^ and myosin-1C^35^ contain 16 and 35 additional amino acids at their N terminus (Fig. 1*B*). The three human myosin-1C splice isoforms are otherwise identical in their structural organization, undergo analogous post-translational modifications, and are capable of interacting with the same partner proteins (12, 13). In rodents and primates, myosin-1C^0^ and myosin-1C^16^ isoforms are ubiquitously produced. In contrast, myosin-1C^35^ shows a tissue-dependent expression profile, suggesting a role in tissue-specific functions (14, 15). Isoform-specific functions of myosin-1C include roles of myosin-1C^16^ and myosin-1C^35^ as nuclear cofactors in chromatin remodeling and transcription activation and a role of myosin-1C^16^ in plasma membrane tension adaptation (3, 6, 16, 17, 18). The underlying regulatory mechanisms that support isoform-specific functional behavior and controlled partitioning between the nucleus and cytoplasm have not been identified. It has been shown that the different myosin-1C isoforms can at least partially complement or replace each other in their function (13). Biochemical studies show that the myosin-1C^0^ isoform produced in rodents is a low-duty-ratio myosin under low-load conditions (19, 20). Biochemical studies on murine myosin-1C^0^ show that external loads increase the duty cycle by means of a force-sensitive mechanism (21).

**Figure 1:**
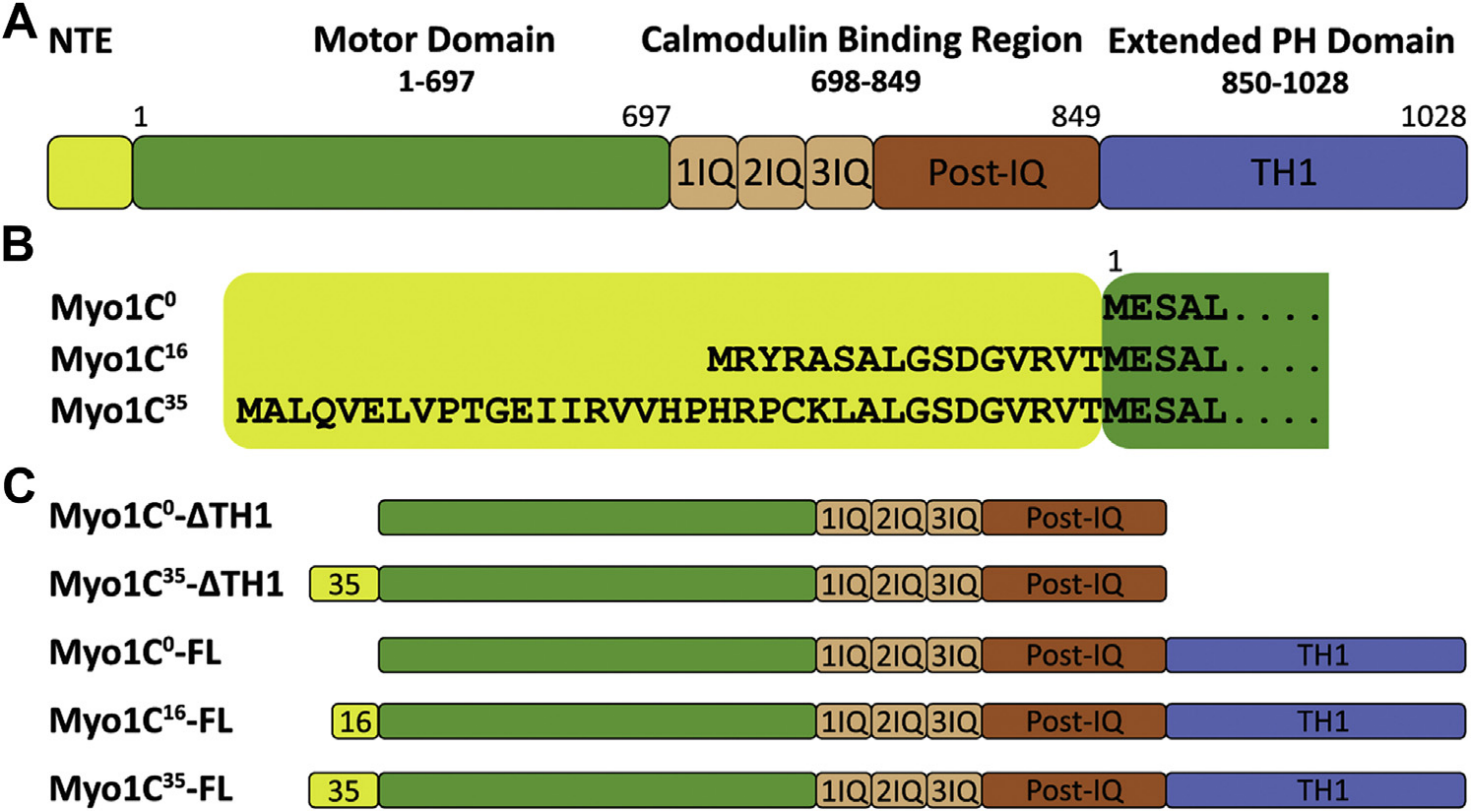
**Schematic representation of the domain structure of human myosin-1C isoforms and of the constructs used in this study**. *A*, domain structure of myosin-1C consisting of a generic myosin motor domain, 3 IQ repeats, a post-IQ domain, and a TH1 domain corresponding to an extended Pleckstrin homology (PH) domain. IQ1 and IQ2 are generic calmodulin-binding motifs. IQ3 and the post-IQ domain together bind a third calmodulin in an unconventional manner, thereby creating a long rigid lever arm region connecting the converter region in the motor domain with the extended PH domain. *B*, sequence alignment showing isoform-specific differences in the N-terminal amino acid sequences of the human myosin-1C splice isoforms. *C*, schematic representation of the myosin-1C constructs used in this study.

In a previous study aimed at dissecting the impact of the 16- and 35-residue NTEs of myosin-1C^16^ and myosin-1C^35^, we described the kinetic properties of the full-length myosin-1C splice isoforms, provided a detailed model of the differential distribution among the isoforms with respect to the close and open state of the actomyosin ADP-bound state during cycling, and related these findings to a structural model where the NTEs form a compact structural domain that crosses the cleft between the converter domain and the calmodulin bound to IQ-repeat 1, thereby enabling a contact between the 35-residue NTE and the relay loop (22). Thus, the NTEs affect the specific nucleotide-binding properties of myosin-1C splice isoforms, adding to their kinetic diversity (22). Here, we describe the isoform-specific changes in the enzymatic and motor properties of the different myosin-1C isoforms by using both full-length and TH1-truncated myosin-1C constructs (Fig. 1*C*). Our results show distinct differences for ADP release, duty cycle, filament sliding velocity, and force-sensing behavior between the isoforms. Furthermore, we observed that in the presence of saturating concentrations of the myosin-1C^35^–derived peptide NTE^35^, the sliding velocity of the Myo1C^0^-ΔTH1·NTE^35^ complex closely resembles that of Myo1C^35^-ΔTH1. The Myo1C^0^-ΔTH1·NTE^16^ complex propelled actin filaments at an intermediate velocity. The changes in motor activity mediated by the different NTEs are consistent with the different roles of myosin-1C isoforms, which range from slow transporter to molecular tension holder (1, 6, 18, 19).

## Results

### Expression and purification of human Myo1C

Constructs for the recombinant production of Myo1C^0^-ΔTH1 and Myo1C^35^-ΔTH1 were coproduced with calmodulin in the baculovirus *Sf9* system and purified to near homogeneity (>95 % purity). Typical yields were 1.6 mg of Myo1C^0^-ΔTH1 and 0.3 mg of Myo1C^35^-ΔTH1 from 2 × 10^9^ *Sf9* cells. The three full-length isoforms of human myosin-1C (Myo1C^0^-FL, Myo1C^16^-FL, and Myo1C^35^-FL) were produced with yields of approximately 0.1 mg of homogeneous protein from 2 × 10^9^ HEK293SF-3F6 cells.

### Isoform-dependent changes in actin-activated ATP turnover

**Figure 2:**
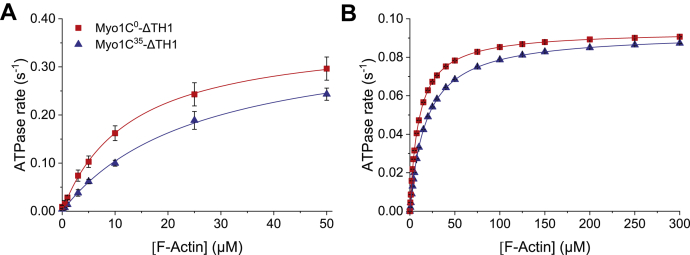
**Isoform-specific differences in actin-activated Mg**^**2+**^**-ATPase activity**. *A*, steady-state actin-activated ATPase activity was measured at 37 °C with actin concentrations in the range from 0 to 50 μM. Error bars represent standard deviations from at least three measurements of each data point. *B*, global fitting simulations of the ATP turnover of Myo1C-ΔTH1 isoforms in the presence of 0 to 300 μM actin and at 20 °C. The parameters *k*_cat_, *K*_app.actin_, and *k*_cat_/*K*_app.actin_ were obtained by fitting the data to the Michaelis–Menten equation. *K*_0.5_ defines *K*_app.actin_, plateau values define *k*_cat_, and *k*_cat_/*K*_app.actin_ is defined by the initial slope of the fit curve at concentrations of actin much lower than *K*_app.actin_. Results are summarized in Table 1.

Basal and actin-activated ATP turnover were initially measured at 37 °C. The rate of ATP turnover in the absence of actin (*k*_basal_) differs approximately 2-fold for Myo1C^0^-ΔTH1 and Myo1C^35^-ΔTH1, with values of 0.009 ± 0.003 s^-1^ and 0.004 ± 0.003 s^-1^, respectively. The actin-activated steady-state ATPase activities of Myo1C^0^-ΔTH1 and Myo1C^35^-ΔTH1 were determined at actin concentrations ranging from 0 to 50 μM and fitting of the data to the Michaelis–Menten equation (Fig. 2*A*). *K*_app.actin_ is the actin concentration at half maximum activation of ATP turnover, and *k*_cat_ corresponds to the maximum value of ATP turnover in the presence of saturating actin concentrations. For both Myo1C^0^-ΔTH1 and Myo1C^35^-ΔTH1, *k*_cat_ corresponds to 0.37 ± 0.01 s^-1^. Compared to the *K*_app.actin_ of 12.7 ± 0.7 μM measured with Myo1C^0^-ΔTH1, the *K*_app.actin_ of Myo1C^35^-ΔTH1 is increased 2-fold to 25.6 ± 1.8 μM (Fig. 2*A* and Table 1).

**Table 1:** Kinetic and mechanical parameters of human Myo1C-ΔTH1

Parameter	Signal and measured parameter	Myo1C^0^-ΔTH1	Myo1C^35^-ΔTH1
Steady-state ATPase (37 °C)			
*k*_basal_ (s^-1^)	NADH assay; *k*_0_	0.009 ± 0.003	0.004 ± 0.003
*K*_app.actin_ (μM)	NADH assay; *K*_0.5_	12.7 ± 0.7	25.6 ± 1.8
*k*_cat_ (s^-1^)	NADH assay; *k*_max_	0.37 ±0.01	0.37 ± 0.01
*k*_cat_/*K*_app.actin_ (μM^-1^ s^-1^)	NADH assay; initial slope	0.024 ± 0.001	0.012 ± 0.001
*K*_app.actin_ (μM) (20 °C)	NADH assay; *K*_0.5_	9.8 ± 0.1	17.9 ± 0.1
*k*_cat_ (s^-1^)	NADH assay; *k*_max_	0.09 ± 0.01	0.09 ± 0.01
*k*_cat_/*K*_app.actin_ (μM^-1^ s^-1^)	NADH assay; initial slope	0.008 ± 0.001	0.005 ± 0.001
Active site isomerization (20 °C)			
*K*_α_	Pyrene-labeled actin; A_fast_/A_slow_	0.90 ± 0.03	3.70 ± 0.20
*k*_+α_ (s^-1^)	Pyrene-labeled actin, *k*_max,slow_	4.1 ± 0.2	3.9 ± 0.2
*k*_-α_ (s^-1^)	*k*_+α_/*K*_α_ (calc.)	4.56 ± 0.13	1.05 ± 0.11
ATP binding (20 °C)			
1/*K*_1_ (μM)	Pyrene-labeled actin, *K*_0.5,fast_	154 ± 31	405 ± 79
*k*_+2_ (s^-1^)	Pyrene-labeled actin, *k*_max,fast_	37.1 ± 1.6	37.0 ± 2.0
*K*_1_*k*_+2_ (μM^-1^ s^-1^)^a^	Pyrene-labeled actin, initial slope	0.16 ± 0.01	0.07 ± 0.01
ATP hydrolysis (20 °C)			
*k*_+3_ + *k*_-3_ (s^-1^)	Tryptophan, *k*_max_	74.6 ± 1.6	75.7 ± 0.8
Actin binding and release (20 °C) (in the absence of nucleotides)			
*k*_+A_ (μM^-1^ s^-1^)^b^	Pyrene-labeled actin, slope	1.46 ± 0.07	2.22 ± 0.08
*k*_-A_ (s^-1^)	Pyrene-labeled actin, *k*_obs_	0.019 ± 0.001	0.037 ± 0.001
*K*_A_ (nM)	*k*_-A_/*k*_+A_ (calc.)	13.7 ± 0.1	16.9 ± 0.2
Phosphate release (20 °C)			
*k*_obs_ (s^-1^)^c^	MDCC-PBP	0.021 ± 0.001	0.010 ± 0.001
*k*_+4_ (s^-1^)	NADH assay, global fit	0.10 ± 0.01	0.10 ± 0.01
ADP binding and release			
*K*_5_ (μM)^d^ (20 °C)	Pyrene-labeled actin, A_slow_/A_total_	0.46 ± 0.08	0.23 ± 0.03
*k*_+5_ (s^-1^) (20°/37 °C)	Pyrene-labeled actin, *k*_min,slow_	1.59 ± 0.07/7.8 ± 0.1	0.87 ± 0.03/3.8 ± 0.1
*k*_*-*5_ (μM^-1^ s^-1^) (20 °C)	*k*_+5_/*K*_5_ (calc.)	3.45 ± 0.75	3.78 ± 0.62
Duty ratio (20 °C)	*k*_+4_/(*k*_+4_ + *k*_+5_) (calc.)	0.044 ± 0.002	0.075 ± 0.001
Motor properties (37 °C)			
Sliding velocity (nm s^-1^)	*In vitro* motility assay	52.1 ± 4.9	14.4 ± 4.2
*k*_f0_ (s^-1^)^e^	Frictional load assay	70.3 ± 3.6	68.2 ± 3.9
*k*_i_ (s^-1^)^e^	Frictional load assay	8.0 ± 0.3	3.7 ± 0.2
*w* (nm)^e^	Frictional load assay	7.8 ± 0.4	3.7 ± 0.1
P_max_ (aW)^f^	Frictional load assay	∼0.05	∼0.003
F_Pmax_ (pN)^f^	Frictional load assay	∼2.0	∼0.45

Stopped-flow buffer and steady-state assay buffer: 25 mM Hepes pH 7.5, 50 mM KCl, 5 mM MgCl_2_, 0.5 mM DTT; Motility assay buffer: 20 mM imidazole pH 7.5, 50 mM KCl, 5 mM MgCl_2_, 2.0 mM EGTA.

a derived from the initial slope of the plot *k*_obs,fast_ versus [ATP].

b derived from the slope of the plot *k*_obs_ versus [actin].

c in the presence of 5 μM F-actin at 20 °C.

d derived from the fit A_slow_/A_total_ = [ADP]/(*K*_5_ + [ADP]).

e derived from Equation 1.

f Based on the evaluation of Figure 8B and on the reported stall force for a single myosin-1C^0^ motor of ∼5 pN (21), we estimate that in our assay approximately 120 motors interact productively per actin filament; single-motor parameters were derived from Equation 2, which was extended by a term representing frictional force.

**Figure 3:**
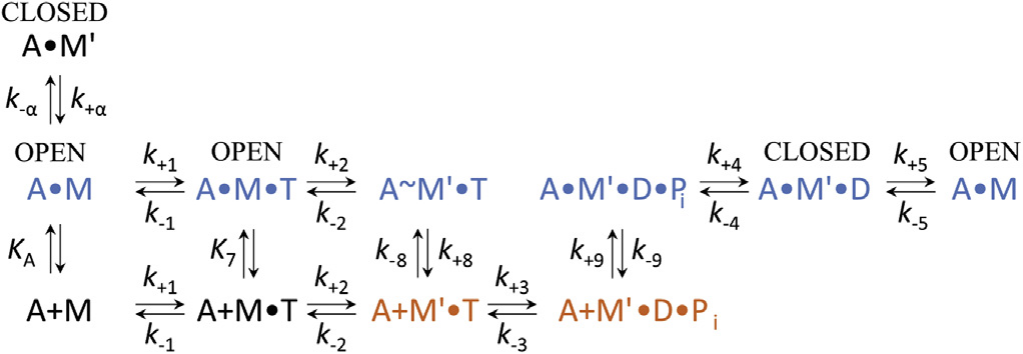
**Minimal kinetic reaction scheme of the acto·myosin-1C ATPase cycle.** The main pathway of myosin-1C is highlighted in *blue* and *orange* indicating Myo1C in the actin-bound and actin-unbound states, respectively. ‘A’ refers to actin, ‘M’ to myosin-1C, ‘T’ to ATP, and ‘D’ to ADP; subscript A refers to actin (*K*_A_); M' refers to closed state; rate constants are written as *k*_+_ for the forward and *k*_−_ for the backward reaction.

To obtain explicit solutions for the mechanism shown in Figure 3, we performed numerical integration by global fitting using rate constants determined in transient kinetic experiments (Table 2). As transient kinetic experiments were performed at 20 °C, we performed additional measurements of actin-activated steady-state ATPase activities at this temperature (Table 1). In addition to the rate constants determined in transient kinetic experiments, we used the experimentally determined values for the apparent second-order rate constant for actin binding (*k*_cat_/*K*_app.actin_) as additional constraints during simulations, as they are well defined by the initial slope of the data fit to the Michaelis–Menten equation at [actin] << *K*_app.actin_ (23). The resulting simulated data set describes the actin dependence of ATP turnover for actin concentrations up to 300 μM (Fig. 2*B*). The global fitting results support a model where *K*_app.actin_ is dominated by the equilibrium constant for actin dissociation from the A·M'·D·P_i_ complex. Simulated modifications probing the role of changes in the rate of ADP release show only negligible effects on *K*_app.actin_.

**Table 2:** Kinetic parameters of human Myo1C-ΔTH1 isoforms obtained by global fit simulation

Individual reaction step	Nomenclature KinTek Explorer	Nomenclature used in this study	Units	Myo1C^0^-ΔTH1	Myo1C^35^-ΔTH1
AM + T **⇌** AMT	k+1	*k*_+1_	μM^-1^ s^-1^	4.0	4.1
	k–1	*k*_-1_	s^-1^	621	1650
	1/K1	1/*K*_1_	μM	156.0	405.0
AMT **⇌** AM′T	k+2	*k*_+2_	s^-1^	37.00	37.00
	k–2	*k*_-2_	s^-1^	1.0	6.9
	K2	*K*_2_		37.0	5.36
AM′T **⇌** M′T + A	k+3	*k*_+8_	s^-1^	10.1	10.1
	k–3	*k*_-8_	μM^-1^ s^-1^	0.01	0.01
M′T **⇌** M′DPi	k+4	*k*_+3_+*k*_-3_	s^-1^	75.00	75.00
M′DPi + A **⇌** AM′DPi	k+5	*k*_+9_	μM^-1^ s^-1^	0.79	1.2
	k–5	*k*_-9_	s^-1^	8.53	26.6
	K5	*K*_9_	μM	10.8	22.2
AM′DPi **⇌** AM′D + Pi	k+6	*k*_+4_	s^-1^	0.10	0.10
	k–6	*k*_-4_	μM^-1^ s^-1^	0.08	0.08
AM′D **⇌** AM + D	k+7	*k*_+5_	s^-1^	1.66	0.86
	k–7	*k*_-5_	μM^-1^ s^-1^	3.94	3.94
	K7	*K*_5_	μM	0.42	0.22
M + A **⇌** AM	k+8	*k*_+A_	μM^-1^ s^-1^	1.47	2.27
	k–8	*k*_-A_	s^-1^	0.019	0.037
	K8	*K*_A_	nM	13.01	16.30
AM′ **⇌** AM	k+9	*k*_+α_	s^-1^	4.2	3.9
	k–9	*k*_-α_	s^-1^	4.72	1.05
	K9	*K*_α_		0.90	3.70

Shown in *red* are the experimentally determined parameters measured by transient kinetic experiments that were used to constrain the simulation. Conditions used were 25 mM Hepes pH 7.5, 50 mM KCl, 5 mM MgCl_2_, 0.5 mM DTT at 20 °C.

### ATP-induced dissociation of myosin-1C from filamentous actin

**Figure 4:**
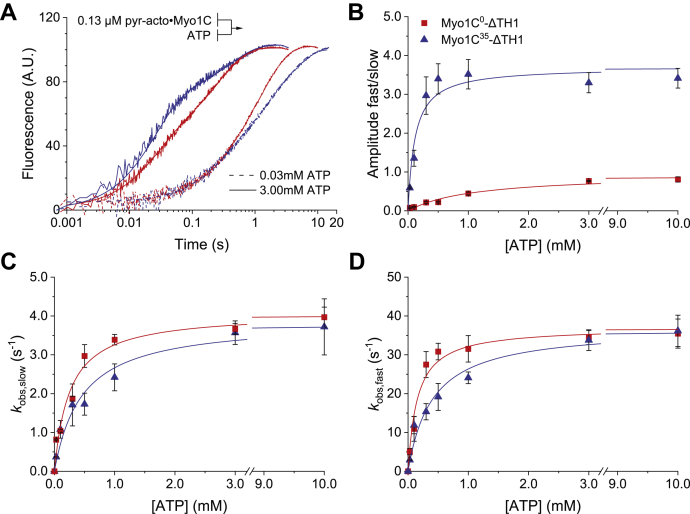
**ATP-induced dissociation of pyrene-labeled acto**·**Myo1C**^**0**^**-ΔTH1 and acto**·**Myo1C**^**35**^**-ΔTH1.***A*, pyrene fluorescence transients observed upon mixing 130 nM acto·Myo1C-ΔTH1 isoforms with 0.03 or 3 mM ATP. All concentrations are given as final concentrations after rapid mixing. The averaged fluorescence transients are best described by double exponentials. *B*, ratio of slow to fast phase amplitudes (A_slow_/A_fast_) plotted against the ATP concentration. The data were fitted to a hyperbola in each case. The respective plateau values define the equilibrium constants for isomerization of the nucleotide-binding pocket *K*_α_. *C*, the dependence of *k*_obs,slow_ on ATP concentration was best fitted with a hyperbola in each case. The plateau values define *k*_+α_, the first-order rate constant for the closed-to-open isomerization of the nucleotide-binding pocket of Myo1C^0^-ΔTH1 and Myo1C^35^-ΔTH1. *D*, Similarly, the dependence of *k*_obs,fast_ on ATP concentration is well described by hyperbolas for both isoforms. The best fits to *k*_obs,fast_ = *K*_1_*k*_+2_[ATP]/(1 + *K*_1_[ATP]) are superimposed. The plateau values define near-identical values for *k*_+2_. The ATP concentration required for half-maximal saturation defines (1/*K*_1_). Here, we observed a 2.6-fold difference between the values obtained for acto·Myo1C^0^-ΔTH1 and acto·Myo1C^35^-ΔTH1. Fitted parameters are summarized in Table 1. Error bars represent standard deviations from at least three determinations of each data point. The experimental curves in panel A correspond to the averaged signals from four independent measurements; A.U., arbitrary units. Lines and symbols are shown in *red* and *blue* for Myo1C^0^-ΔTH1 and Myo1C^35^-ΔTH1, respectively.

The dissociation of Myo1C^0^-ΔTH1 and Myo1C^35^-ΔTH1 from pyrene-labelled F-actin by ATP is accompanied by a biphasic increase in the fluorescence signal (Fig. 4*A*). The reaction is best fitted by two exponentials and was analyzed according to the model shown in Figure 3 (19, 24). The equilibrium constant K_α_ for the transition from the A·M to A·M' state is given by the ratio of fast to slow phase amplitude at saturating ATP concentrations (24). *K*_α_ was determined with values of 0.90 ± 0.03 for Myo1C^0^-ΔTH1 and 3.70 ± 0.20 for Myo1C^35^-ΔTH1 (Fig. 4*B*).

The observed rate constants for the slow phase have a hyperbolic dependence on ATP concentration (Fig. 4*C*). The fit curves converge toward plateau values that define the isomerization rate *k*_+α_ for the nucleotide-binding pockets of Myo1C^0^-ΔTH1 (4.1 ± 0.2 s^-1^) and Myo1C^35^-ΔTH1 (3.9 ± 0.2 s^-1^).

The observed rate constants for the fast phase were linearly dependent upon ATP concentrations in the range of 5 to 50 μM. The apparent second-order rate constants for ATP binding *K*_1_*k*_*+*2_ are defined by the respective slopes. *K*_1_*k*_*+*2_ is 2.4-fold reduced for Myo1C^35^-ΔTH1 compared with Myo1C^0^-ΔTH1 with values of 0.068 ± 0.002 μM^-1^ s^-1^ and 0.162 ± 0.008 μM^-1^ s^-1^, respectively. At high ATP concentrations (>2 mM), the observed rate constants saturate, and the [ATP] dependence of *k*_obs_ is described by a hyperbola as predicted by Figure 3, where *k*_max_ = *k*_+2_ and *K*_0.5_ = 1/*K*_1_ (Fig. 4*D*). In the case of Myo1C^0^-ΔTH1, the affinity of ATP for the actin–myosin complex 1/*K*_1_ was determined as 154 ± 31 μM for Myo1C^35^-ΔTH1 and as 405 ± 79 μM for Myo1C^35^-ΔTH1. The rate constant *k*_+2_ for the isomerization that limits the conformational change from high to low actin affinity equals 37.1 ± 1.6 s^-1^ for Myo1C^0^-ΔTH1 and 37.0 ± 2.0 s^−1^ Myo1C^35^-ΔTH1.

### Isoform-specific changes in ADP binding to acto·myosin-1C

**Figure 5:**
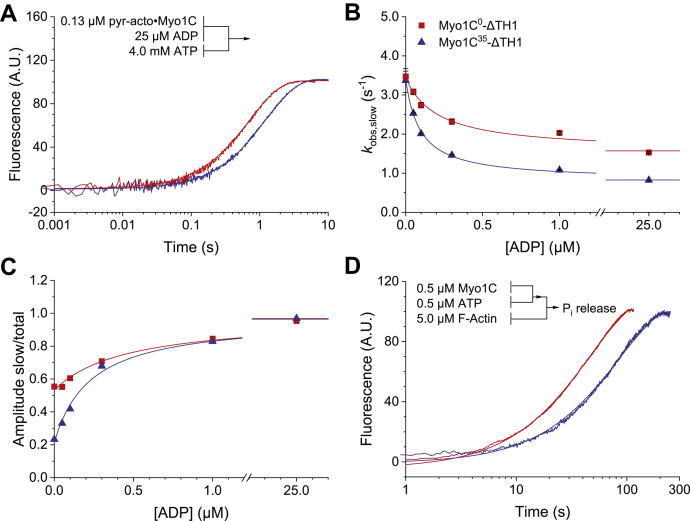
**Interaction of Myo1C**^**0**^**-ΔTH1 and Myo1C**^**35**^**-ΔTH1 with the hydrolysis products ADP and P**_**i**_**in the presence of pyrene-labeled actin**. *A*, inhibition of the ATP-induced dissociation of pyrene-acto·Myo1C by ADP. The observed increases in fluorescence are best described by double exponentials yielding values for *k*_obs,slow_, *k*_obs,fast_, A_slow_, and A_fast_. *B*, the dependence of *k*_obs,slow_ on ADP concentration was best fitted with a hyperbola. The apparent affinities for ADP correspond to 0.21 ± 0.06 μM and 0.10 ± 0.03 μM for acto·Myo1C^0^ and acto·Myo1C^35^. The minimum observed rates in the presence of saturating ADP concentrations define the rate constant for ADP dissociation (*k*_+5_). *C*, plot of the fraction of A_slow_ against [ADP]. The data were fitted to a hyperbola with A_slow_/A_total_ = [ADP]/(*K*_5_ + [ADP]). *D*, P_i_ release from acto·Myo1C was followed in a double mixing experiment with 1.8 μM of the phosphate sensor MDCC-PBP and 5 μM F-actin after mixing. The averaged fluorescence transients are best described by single exponentials yielding an apparent rate constant for phosphate release. Fitted parameters are summarized in Table 1. All concentrations are given as final concentrations after rapid mixing. Error bars represent standard deviations from at least three determinations of each data point. The experimental curves in panel A and D correspond to the averaged signals from four independent measurements. Lines and symbols are shown in *red* and *blue* for Myo1C^0^-ΔTH1 and Myo1C^35^-ΔTH1, respectively.

To measure ADP release kinetics from the acto· Myo1C-ΔTH1 constructs, we preincubated the protein with ADP and determined the rate of displacement of ADP by monitoring the biphasic exponential increase of the pyrene fluorescence signal that follows the addition of excess ATP (Fig. 5*A*). Since ADP is in rapid equilibrium with A·M and A·M' on the time scale of the slow phase of the reaction, the *k*_obs_ of the slow phase decreases with a hyperbolic dependence on the concentration of ADP (19). The fit of *k*_obs,slow_ converges toward a minimal plateau value that defines the rate constant for ADP release (*k*_+5_) with values of 1.59 ± 0.07 s^−1^ and 0.87 ± 0.03 s^−1^ for acto·Myo1C^0^-ΔTH1 and acto·Myo1C^35^-ΔTH1, respectively (Fig. 5*B*). The ADP concentrations at which half-saturation is reached define the apparent ADP affinity constant *K*_app_ with values of 0.21 ± 0.06 μM for acto·Myo1C^0^ and 0.10 ± 0.03 μM for acto·Myo1C^35^. The relationship between *K*_5_, the dissociation equilibrium constant for ADP to acto·Myo1C, and the apparent equilibrium constant for ADP is defined by *K*_app_ = (*K*_5_^−^/^−^(1 + 1/*K*_α_). The resulting calculated *K*_5_ values correspond to 0.44 ± 0.12 μM for acto·Myo1C^0^ and 0.13 ± 0.04 μM for acto·Myo1C^35^. Plots of the fraction of A_slow_ as a function of [ADP] show a hyperbolic dependence, which at half-saturation directly defines *K*_5_ giving values of 0.46 ± 0.08 μM for Myo1C^0^-ΔTH1 and 0.23 ± 0.03 μM for Myo1C^35^-ΔTH1 with a smaller margin of error than the calculated values (Fig. 5*C*). The second-order rate constants for ADP binding (k_−5_) were calculated from *k*_+5_/*K*_5_, yielding values of 3.45 ± 0.75 μM^-1^ s^-1^ and 3.78 ± 0.62 μM^-1^ s^-1^ for acto·Myo1C^0^-ΔTH1 and acto·Myo1C^35^-ΔTH1, respectively (Table 1).

### Isoform-specific changes affecting P_i_ release from acto·myosin-1C

We measured the P_i_ release kinetics for the myosin-1C isoforms in the presence of 5 μM actin (Fig. 5*D*). The observed rates of P_i_ release were 0.021 ± 0.001 s^−1^ from acto·Myo1C^0^-ΔTH1 and 0.010 ± 0.001 s^−1^ from acto·Myo1C^35^-ΔTH1. Considering that ATP-turnover measurements in the presence of 5 μM actin, performed at 20 °C and under identical buffer conditions, showed only 15 and 30% of the maximum activation level for Myo1C^35^-ΔTH1 and Myo1C^0^-ΔTH1, respectively, we estimate that both constructs share a maximum rate of P_i_ release of about 0.09 s^−1^, which limits the rate of ATP turnover. These estimates are in good agreement with values of 0.10 ± 0.01 s^−1^ for *k*_+4_, the rate constants for P_i_ release in the presence of saturating concentrations of actin, obtained for both constructs by global fitting simulation (Table 1).

### Binding of myosin-1C isoforms to F-actin

**Figure 6:**
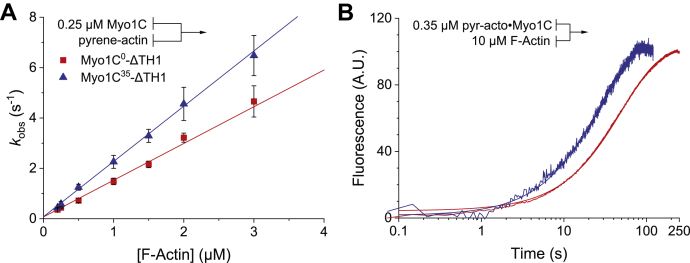
**Actin binding and release in the absence of nucleotides.** Binding of the Myo1C-ΔTH1 constructs to pyrene-labeled F-actin quenches the pyrene fluorescence. The transients obtained upon mixing a Myo1C-ΔTH1 construct with pyrene-labeled actin in a stopped-flow spectrophotometer follow a mono-exponential. *A*, the observed rate constants show a linear dependence on the concentration of pyrene-labeled actin in the range from 0.25 to 3.0 μM. The second-order rate constants for binding to actin (*k*_+A_) is derived from the slopes. *B*, fluorescence transients observed after chasing pyrene-labeld actin from the pyrene–actomyosin complex with excess F-actin. The observed processes could be fit to single exponentials where *k*_obs_ corresponds directly to the rates of actin dissociation (*k*_-A_). The fitted parameters are summarized in Table 1. All concentrations are given as final concentrations after rapid mixing. Error bars in panel A represent standard deviations from at least three determinations of each data point; the experimental curves in panel B correspond to the averaged signals from four independent measurements. Lines and symbols are shown in *red* and *blue* for Myo1C^0^-ΔTH1 and Myo1C^35^-ΔTH1, respectively.

The rate of myosin-1C binding to actin filaments *k*_+A_ was measured by recording the exponential decrease of the pyrene fluorescence signal that follows rapid mixing of the proteins. Secondary plots of the observed rate constants against the actin concentration (0.25–3.0 μM) show linear dependencies (Fig. 6*A*). The second-order association rate constants *k*_+A_ are defined by the slope of the fit lines. In comparison with Myo1C^0^-ΔTH1, *k*_+A_ is 1.5-fold increased for Myo1C^35^-ΔTH1. The dissociation rate constant *k*_-A_ was determined by chasing pyrene-labeled actin with a large excess of unlabeled actin. Figure 6*B* shows the time course for displacement of pyrene-labeled actin from 0.35 μM pyrene-acto·Myo1C-ΔTH1 by the addition of 10 μM unlabeled actin. The time dependence of the ensuing rise in fluorescence amplitude is best described by a single-exponential function, where *k*_obs_ corresponds directly to the dissociation rate constant *k*_-A_. Our results show a 2-fold slower rate of Myo1C^0^-ΔTH1 dissociation from F-actin than that of Myo1C^35^-ΔTH1. The equilibrium dissociation constant *K*_A_ for the interaction of the myosin-1C isoforms with F-actin in the absence of ATP was calculated from the ratio of the rate constants *k*_-A_/*k*_+A_. *K*_A_ corresponds to 13.7 ± 0.1 nM for Myo1C^0^-ΔTH1 and 16.9 ± 0.2 nM in the case of Myo1C^35^-ΔTH1 (Table 1).

### Isoform-specific changes in the motility of human myosin-1C isoforms

**Figure 7:**
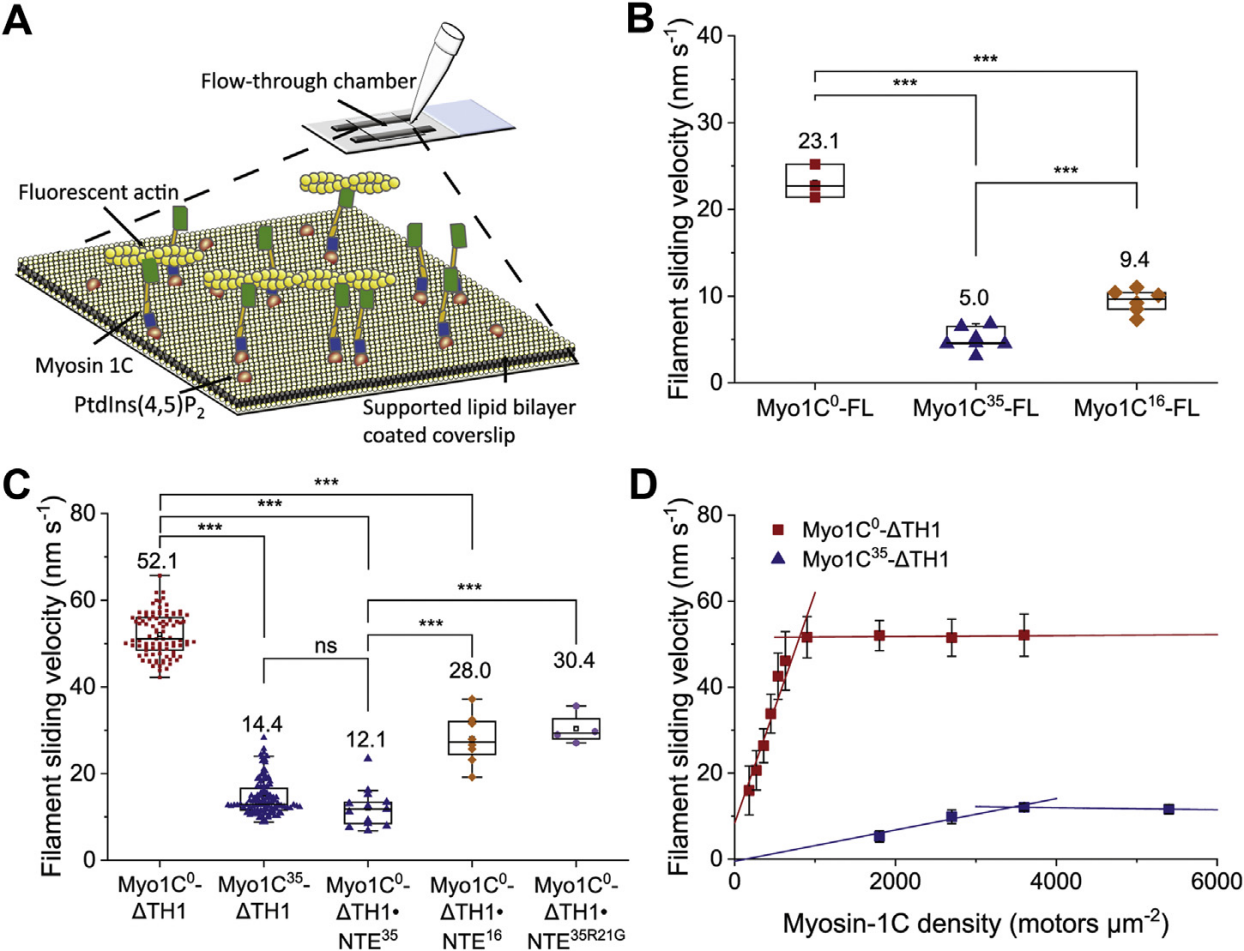
**Isoform-specific changes of myosin-1C motor activity.***A*, schematic illustration of the flow cell coated with a supported lipid bilayer containing PtdIns(4,5)P_2_ (SLP)_._ The SLP is spread on a glass coverslip, where it facilitates attachment of Myo1C-FL via the extended PH domain (*blue*). Motor domains (*green*) are optimally oriented for productive interactions with TRITC-phalloidin–labelled actin filaments (*yellow*). Following the addition of ATP, the actin filaments move in a unidirectional fashion. *B*, Box-and-whisker diagram of averaged filament sliding velocities for Myo1C^0^-FL, Myo1C^35^-FL, and Myo1C^16^-FL. *C,* Box-and-whisker diagram of the average filament sliding velocities of Myo1C^0^-ΔTH1 and Myo1C^35^-ΔTH1 are shown on the left side of the panel. Results obtained for Myo1C^0^-ΔTH1 in the presence of peptides NTE^35^, NTE^16^, and NTE^35R21G^ are shown on the right side of the panel. Each data point in the box-and-whisker diagram represents the averaged filament sliding velocity determined using an independent flow cell and analyzing more than 100 unidirectional trajectories. *D*, Myosin-1C isoform-specific changes of actin filament sliding velocity measured at various myosin-1C surface densities in the range from 180 to 5400 motors μm^-2^. The motor densities required to reach the maximum velocity correspond to about 900 and 3600 motors μm^-2^ for Myo1C^0^-ΔTH1 and Myo1C^35^-ΔTH1, respectively. The error bars associated with each data point represent standard deviations from at least three *in vitro* motility measurements, each analyzing more than 100 unidirectional trajectories. Statistical significance was assessed by Student’s paired *t* test (2-tailed) and is assigned as follows: ∗ (*p* < 0.05); ∗∗ (*p* < 0.01); ∗∗∗ (*p* < 0.001). Lines and symbols are shown in *red* for Myo1C^0^, *orange* for Myo1C^16^, and *blue* for Myo1C^35^ constructs. TRITC, tetramethylrhodamine.

To determine the influence of the NTE peptides on myosin-1C motor function, we performed *in vitro* motility assays. In particular, we analyzed isoform-specific differences in the motile activity of Myo1C-FL constructs on supported planar lipid bilayers containing 2% phosphatidyl-inositol-4,5-bisphosphate (PtdIns(4,5)P_2_) and 98% dioleoylphosphocholine (Fig. 7*A*). All three full-length constructs displayed smooth and continuous movement when flow-cell loading concentrations of 1 μM Myo1C-FL construct or greater were used. The maximum sliding velocities of Myo1C^0^-FL, Myo1C^16^-FL, and Myo1C^35^-FL are 23.1 ± 1.9 nm s^-1^, 9.4 ± 1.4 nm s^-1^, and 5.0 ± 1.3 nm s^-1^, respectively, under these conditions (Fig. 7*B* and Table 3).

**Table 3:** Kinetic and functional properties of human full-length myosin-1C

Parameter	Myo1C^0^-FL	Myo1C^16^-FL	Myo1C^35^-FL
Steady-state ATPase			
*k*_cat_ (s^-1^) Π (20 °C)	0.10 ± 0.01	0.12 ± 0.02	0.10 ± 0.01
ADP release			
*k*_+5_ (s^-1^) Π (20 °C)	0.70 ± 0.10	0.50 ± 0.02	0.40 ± 0.03
Duty ratio (20 °C)	0.12 ± 0.02	0.19 ± 0.04	0.20 ± 0.03
Motor properties			
Sliding velocity (nm s^−1^)Ω (37 °C)	23.1 ± 1.9	9.4 ± 1.4	5.0 ± 1.3

Π Data from (22) measured in 20 mM Mops pH 7.0, 50 mM potassium acetate, 2 mM ATP, 2 mM MgCl_2_, 0.2 mM EGTA, 1 mM DTT, 20 °C.

Ω 25 mM Hepes pH 7.5, 100 mM KCl, 0.5 mM MgCl_2_, 37 °C.

The observed isoform-dependent changes in the sliding velocity of the Myo1C-FL constructs are consistent with the results obtained for the Myo1C-ΔTH1 constructs with surface attachment via antibodies directed against the C-terminal histidine tag of these constructs. In the case of the TH1-truncated constructs, we observed approximately 4-fold differences for both the number of myosin motors required to support continuous smooth movement of actin filaments and the maximal sliding velocity of the constructs. We observed a linear dependence between the Myo1C^0^-ΔTH surface density and the observed velocity over the range of 200 to 900 motors μm^-2^. At surface densities greater than 900 motors μm^-2^, a plateau value of 52.1 ± 4.9 nm s^−1^ is reached. In contrast, smooth, continuous movement of actin filaments on lawns of Myo1C^35^-ΔTH requires at least 1800 motors μm^-2^. The plateau value of 14.4 ± 4.2 nm s^−1^ is reached only at surface concentrations greater 3600 Myo1C^35^-ΔTH motors μm^-2^ (Fig. 7, *C*–*D*). The linear dependence between Myo1C^35^-ΔTH surface density and velocity has a slope 16-fold smaller than that observed for the short isoform (Fig. 7*D*). With the exception of murine construct Myo1C^0^-1IQ-SAH which features a stable single α-helix lever-arm extension and supports ∼10-fold faster velocities (25), the sliding velocities of the actin filament observed for human Myo1C^0^-ΔTH1 and Myo1C^0^-FL are in good agreement with those previously reported for equivalent murine constructs (Table 4) (26, 27, 28).

**Table 4:** Comparison of kinetic and functional parameters of human and murine myosin-1C constructs lacking the extended PH domain

Reference	aThis study		bAdamek et al., 2011 (25)	c Greenberg et al., 2012 (21)	c Greenberg et al., 2015 (28)
Organism	Human		Mouse	Mouse	Mouse
Experimental temperature	^#^20 °C/^§^37 °C		^#^20 °C/^Π^ room temp./^§^37 °C	^#^20 °C/^§^37 °C	^#^20 °C/^§^37 °C
Construct name	Myo1C^0^-ΔTH1		Myo1C^0^-1IQ-SAH	Myo1C^0^-3IQ	Myo1C^0^-3IQΔN
Steady-state ATPase	#	§	§	#	
*k*_cat_ (s^-1^)	0.09 ± 0.01	0.37 ±0.01	0.66 ± 0.35	n.d.	n.d.
*K*_app.actin_ (μM)	9.8 ± 0.1	12.7 ± 0.7	17.50 ± 27.85	n.d.	n.d.
*k*_cat_/*K*_app.actin_ (μM^-1^ s^-1^)	0.008 ± 0.001	0.024 ± 0.001	0.026 ± 0.01^d^	0.0046 ± 0.0006	n.d.
Active site isomerization	#		#	#	#
*k*_+α_ (s^-1^)	4.1 ± 0.2		2.0	4.00 ± 0.03	56 ± 3.2
*k*_-α_ (s^-1^)	4.56 ± 0.13		18.2	12 ± 1	32 ± 4.7
*K*_α_	0.90 ± 0.03		0.11	0.33 ± 0.03	1.8 ± 0.25
ATP binding	#		#	#	#
*k*_+2_ (s^-1^)	37.1 ± 1.6		41.2	26.0 ± 0.8	160 ± 4.6
1/*K*_1_ (μM)	154 ± 31		507	97 ± 15	450 ± 48
*K*_1_*k*_+2_ (μM^-1^ s^-1^)	0.16 ± 0.01		0.081	0.26 ± 0.014	0.35 ± 0.039
Actin binding and release	#			#	
*k*_+A_ (μM^-1^ s^-1^)	1.46 ± 0.07		n.d.	3.4 ± 0.2	n.d.
*k*_-A_ (s^-1^)	0.019 ± 0.001		n.d.	0.0011	n.d.
*K*_A_ (nM)	13.7 ± 0.1		n.d.	0.29	n.d.
Phosphate release	#				
*k*_+4_ (s^-1^)	0.10 ± 0.01		n.d.	n.d.	n.d.
ADP release	#		#	#	#
*k*_+5_ (s^-1^)	1.59 ± 0.07		1.9	3.90 ± 0.06	4.2 ± 0.2
Motor properties	§		Π	§	§
Sliding velocity (nm s^-1^)	52.1 ± 4.9		550 ± 170∗	83 ± 5.9	60 ± 4.6
	§			#	
P_max_ (aW)	∼0.05		n.d.	∼0.008	n.d.

a Stopped-flow buffer and steady-state assay buffer: 25 mM Hepes pH 7.5, 50 mM KCl, 5 mM MgCl_2_, 0.5 mM DTT, 20 °C; motility assay buffer: 20 mM imidazole pH 7.5, 50 mM KCl, 5 mM MgCl_2_, 2.0 mM EGTA, 37 °C.

b Stopped-flow buffer: 20 mM Mops pH7.0, 100 mM KCl, 5 mM MgCl_2_, 1 mM EGTA, 20 °C; steady-state ATPase buffer: 10 mM Tris-HCl pH 7.5, 50 mM, KCl, and 1 mM MgCl_2_, 37 °C (25); motility assay buffer: 25 mM imidazole pH 7.5, 25 mM KCl, 4 mM, MgCl_2_, 1 mM EGTA, room temperature; ∗extended SAH lever arm.

c Stopped-flow buffer and steady-state assay buffer: 10 mM Mops pH 7.0, 25 mM KCl, 1 mM MgCl_2_, 1 mM EGTA, 1 mM DTT, 1 μM CaM, 20 °C; motility assay buffer: 25 mM imidazole pH 7.5, 25 mM KCl, 4 mM MgCl_2_, 1 mM EGTA, 37 °C; optical trap buffer: 10 mM Mops pH 7.0, 25 mM KCl, 1 mM MgCl_2_, 1 mM EGTA, 5 mM DTT, 20 °C (21, 28).

d Determined from the initial slope of the graph.

We have previously reported that in the presence of saturating concentrations of peptide NTE^35^, Myo1C^0^-FL shows the same kinetic behavior in terms of ATP turnover as Myo1C^35^-FL (22). Here, we report a similar ∼4-fold decrease in the filament sliding velocity from 52.1 ± 4.9 nm s^−1^ to 12.1 ± 4.3 nm s^−1^ for acto·Myo1C^0^-ΔTH1 in the presence saturating concentrations of peptide NTE^35^ and an ∼2-fold decrease to 28.0 ± 5.3 nm s^-1^ in the presence of peptide NTE^16^. To test a structural model that predicts a critical contact between residue R21 of the NTE and residue E469 in the relay loop (22), we performed additional assays with Myo1C^0^-ΔTH1 in the presence of peptide NTE^35R21G^. The observed reduction in the sliding velocity to 30.4 ± 3.2 nm s^−1^ is similar to the reduction brought about by peptide NTE^16^. This result supports our model (22) whereby a contact between NTE residue Arg-21 and relay loop residue Glu-469 contributes to defining the functional properties of myosin-1C^35^ (Fig. 7*C*).

### Isoform-dependent modulation of force generation by myosin-1C ensembles

**Figure 8:**
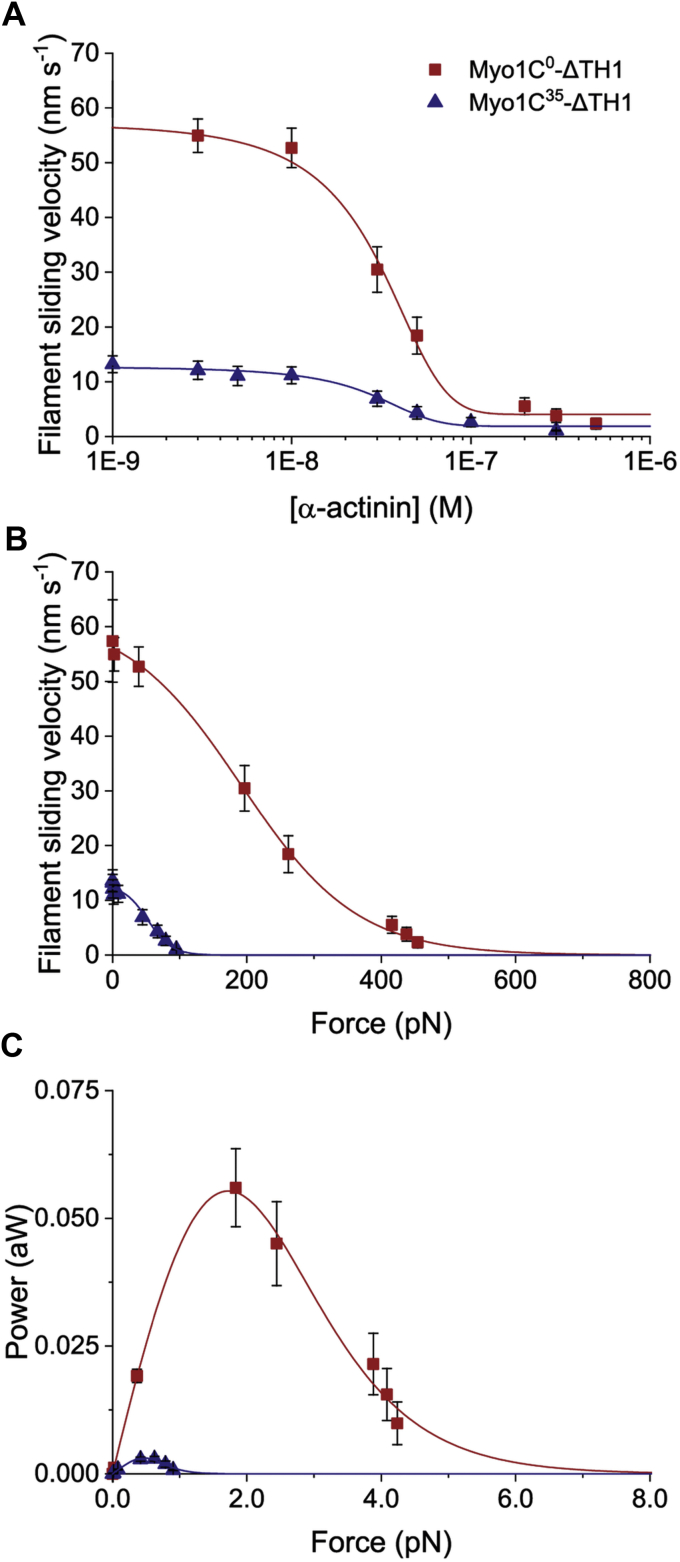
**Differences between Myo1C**^**0**^**-ΔTH1 and Myo1C**^**35**^**-ΔTH1 in force generation and power output.** The mechanochemical behavior of Myo1C^0^ and Myo1C^35^ was compared using a frictional loading assay (29). *A*, the filament sliding velocity of F-actin driven by Myo1C-ΔTH1 is reduced by the addition of α-actinin, generating a resistive force that increases with increasing α-actinin concentrations. The fit curves shown were obtained using the equation$\;\text{y }=\text{ A}2+\left(\text{A}1-\text{A}2\right)/\left(1+{\text{e}}^{\left(\left(\text{x}-{\text{x}}_{0}\right)/\text{dx}\right)}\right)$. The values obtained for the midpoint of the transition and the steepness of the transition correspond to 25 ± 8 nM and 18 ± 4 nM for Myo1C^0^-ΔTH1 and 24 ± 11 nM and 15 ± 7 nM for Myo1C^35^-ΔTH1. *B*, dependence of the observed filament sliding velocity on resistive force. To relate α-actinin concentration to resistive force per filament, we used Equation 1. The fit curves shown were obtained using Equation 2 (21, 28). The values for the force-independent rate (*k*_i_) and the force-dependent rate in the absence of external loads (*k*_f0_) shown in Table 1 were obtained by iterative fitting cycles. *C,* plot of power output against the resistive force. Based on the result shown in panel B and on the reported stall force for a single myosin-1C^0^ motor of ∼5 pN (21), we estimate that in our assay, approximately 120 Myo1C^0^-ΔTH1 motors interact productively per actin filaments. Given that with the exception of the construct used all experimental parameters are identical for the experiments performed with Myo1C^0^-ΔTH1 and Myo1C^35^-ΔTH1, we conclude that for Myo1C^35^-ΔTH1, the stall force is approximately 4-fold reduced. The power output of a single motor was calculated from the product of the filament sliding velocity and the corresponding frictional force. To determine the maximum power output (P_max_) of a single motor and the force where the power output reaches its peak (F_Pmax_), the data were fitted to Equation 2 extended by a term representing frictional force. Lines and symbols are shown in *red* and *blue* for Myo1C^0^-ΔTH1 and Myo1C^35^-ΔTH1, respectively.

To analyze the influence of the NTE peptides on force development, we determined the ability of the different isoforms to move actin filaments in the presence of an external load using frictional loading experiments (29). Binding of surface-attached α-actinin to actin filaments counteracts the driving force of myosin and leads to a reduction in the filament sliding velocity as the external load increases with the concentration of bound α-actinin (Fig. 8*A*). We observed that the load-dependent changes in the sliding velocities of myosin-1C isoforms are best described by a tension-sensing mechanism, as previously derived by Ostap and colleagues using single-molecule measurements (21, 28). The resulting model predicts two sequential transitions for the entire range of loads. A force-dependent and a force-independent transition with associated rate constants *k*_f_(F) and *k*_i_. By fitting the data for the force–velocity dependence of the constructs shown in Figure 8*B* to Equation 1 and assuming working stroke displacements (*w*) of 7.8 nm and 3.7 nm for Myo1C^0^-ΔTH1 and Myo1C^35^-ΔTH1, respectively, we obtained values of 70.3 ± 3.6 s^−1^ for the detachment rate in the absence of external loads (*k*_f0_) and 8.0 ± 0.3 s^−1^ for the force-independent detachment rate (*k*_i_) for Myo1C^0^-ΔTH1. The values obtained for Myo1C^35^-ΔTH1 are 68.2 ± 3.9 s^−1^ and 3.7 ± 0.2 s^−1^. The calculated *k*_i_ values for Myo1C^0^-ΔTH1 and Myo1C^35^-ΔTH1 are similar to the rates of unloaded ADP release (*k*_+5_) measured at 37 °C, with values 7.8 ± 0.1 s^−1^ for Myo1C^0^-ΔTH1 and 3.8 ± 0.1 s^−1^ for Myo1C^35^-ΔTH1. To obtain information about the maximum power output per myosin-1C motor (P_max_) and the force at which maximum power output (F_Pmax_) occurs from our ensemble measurements, we calculated the force–power relationships for Myo1C^0^-ΔTH1 and Myo1C^35^-ΔTH1 by extending Equation 1 with a force term and by estimating the number of interacting motors. The resulting bell-shaped curves define F_Pmax_ and P_max_ values for single motors of ∼2.0 pN and ∼0.05 aW for Myo1C^0^-ΔTH1 and ∼0.45 pN and ∼0.003 aW for Myo1C^35^-ΔTH1, respectively (Fig. 8*C*). The F_Pmax_ value obtained for Myo1C^0^-ΔTH1 is in good agreement with the value of 2.3 pN determined for a similar myosin-1C^0^ construct by single molecule optical trapping, while the F_Pmax_ determined for Myo1C^35^-ΔTH1 is similar to the value of 0.6 pN measured with myosin-1B in single-molecule experiments (21, 30).

## Discussion

Human myosin-1C remains the only myosin for which high-resolution structural information is available that covers the entire molecule. The model of the full-length myosin-1C structure can be obtained by combining the crystal structures of the motor and neck regions (PDB accession code 4BYF) with that of the neck and tail regions of myosin-1C^0^ (PDB accession code 4R8G) (10, 31). The availability of detailed structural information for the whole protein greatly facilitates the generation of constructs that are suitable for studying specific aspects of myosin-1C function. In a previous study, we described how splicing of the human *MYO1C* gene and the resulting changes in the NTR of myosin-1C fine-tune the kinetics of the full-length isoforms of the protein (22). Here, we extend the characterization of functional differences between the myosin-1C isoforms with studies investigating the motile properties of the full-length proteins and the kinetic and mechanochemical properties of engineered constructs that have their C-terminal TH1 domain replaced by an octa-histidine tag. Acceptable yields of the full-length versions of the myosin-1C isoforms were only obtained using homologous expression in suspension-adapted HEK293SF-3F6 cells. In contrast, the TH1-truncated versions can be produced and purified in larger quantities using baculovirus-driven protein production in insect cells. Like many other tail-truncated myosins (31, 32, 33, 34), the TH1-truncated versions retain the actin- and nucleotide-binding properties of the full-length myosin and are therefore more readily available for detailed mechanochemical studies of enzymatic and motor functions. A comparison of results obtained with human and murine myosin-1C^0^ constructs (Table 4) reveals differences of similar magnitude as observed between the murine constructs with the truncated tail (21, 22, 25, 27, 28).

The mechanism of isoform-dependent mechanochemical tuning of myosin-1C is different from that of myosin-1B, which is alternatively spliced in its calmodulin-binding domain, yielding proteins with lever arms of different lengths. Additional differences are stemming from differences in the load dependence of product release and in particular the release of ADP (19, 21, 24, 35, 36, 37). Similarly, this seems to be a major difference between the myosin-1C isoforms and a chimeric construct with myosin-1B–like load-sensing behavior, obtained by replacing 11 N-terminal residues of myosin-1C^0^ with 15 N-terminal residues of myosin-1B (28). The replaced residues are an integral part of the upper 50-kDa domain of myosin-1C and not part of a small independent NTE (28).

**Figure 9:**
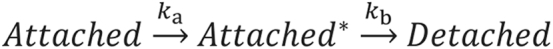
**Model describing the biphasic ATP-induced detachment of acto•myosin-1C.** The model defines the detachment of acto•myosin-1C as the result of a force-independent step and a force-dependent step, but the order of the steps cannot be distinguished by the model alone.

The presence of the NTE^16^ and NTE^35^ peptides in *trans* or covalently attached to the myosin-1C motor domain leads to large changes in load-sensing behavior, including the 18-fold reduction in P_max_ observed for myosin-1C^35^. According to results obtained using optical trap measurements, the detachment of myosin-1C^0^ in the presence of external loads is best described by a two-step process involving a force-dependent transition *k*_f0_ and a force-independent transition *k*_i_ (Fig. 9) (21). The rates observed for *k*_f0_ and *k*_i_ were reported to be consistent with the transitions that limit ATP-induced dissociation at saturating [ATP] *k*_+2_ and the rate of ADP release *k*_+5_, respectively (28). The rate constants for the force-dependent detachment of acto·Myo1C have been shown to undergo only marginal changes in the presence of ADP (21). On the basis of this finding, the frictional loading experiments appear to be suitable for obtaining reasonable estimates of the rate constants *k*_i_ and *k*_f0_. This is supported by the fact that the load-dependent changes in the sliding velocities of all myosin-1C isoforms in our ensemble measurements are best described by the same model. A more detailed analysis of the force-sensing mechanism of myosin-1C^35^ requires single-molecule optical trap measurements.

**Figure 10:**
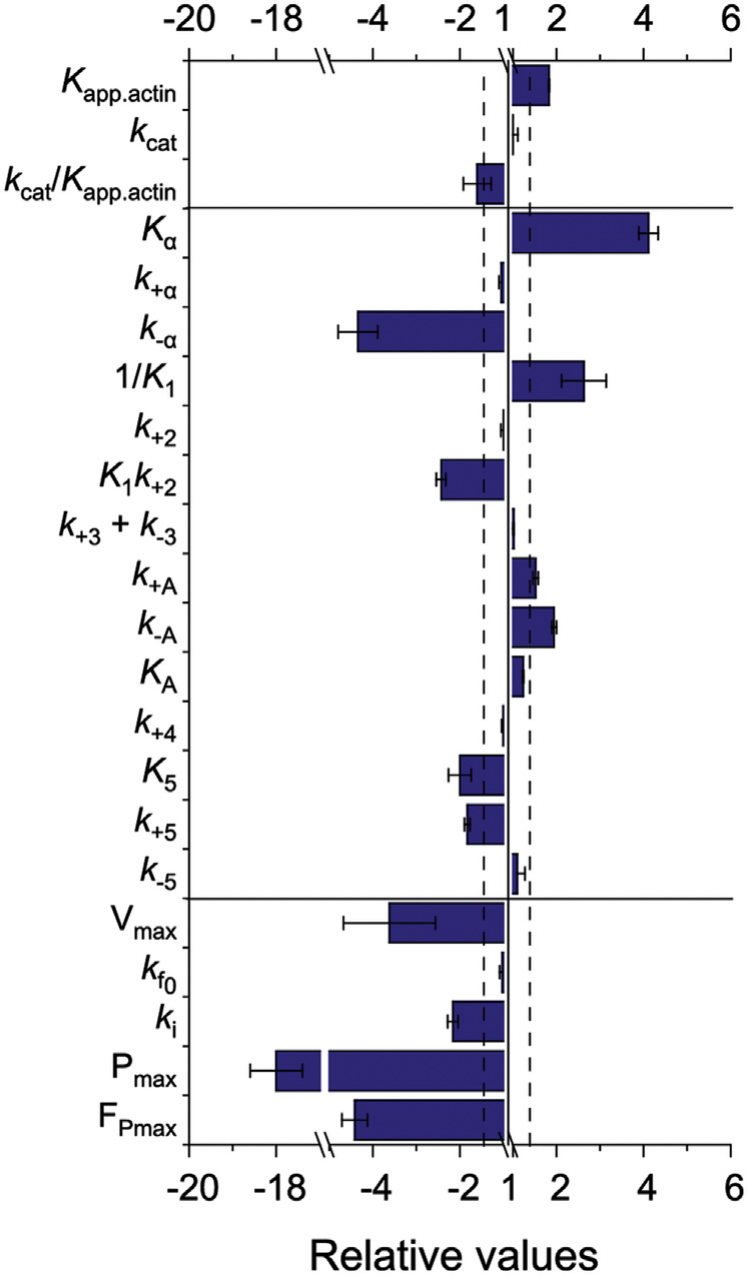
**Comparison of kinetic and functional properties of myosin-1 isoforms.** The graph shows the values obtained for myosin-1 C^35^ relative to those obtained for myosin-1 C^0^; n-fold larger values are positive, and n-fold smaller values are negative. The results are grouped in parameters determined using (*i*) steady-state ATPase measurements, (*ii*) stopped-flow measurements, and (*iii*) *in vitro* motility assays. All parameters were measured under zero-load conditions, with the exception of P_max_ and F_Pmax_. The dashed lines represent 30% deviation from 1.0.

Under low-load conditions, isoform-dependent changes have no or only minor effects on the rates of ATP hydrolysis (*k*_+3_ + *k*_-3_), P_i_ release (*k*_+4_), and ATP turnover (*k*_cat_) (Fig. 10 and Table 1). The major differences between the human myosin-1C isoforms are observed for steps that affect ADP release including the isomerization of the active site pocket ((22) and this study). ADP release contributes to the power stroke of class I myosins and plays a key role in defining the mechanical–chemical properties of these myosins (30, 38, 39). A 2-fold faster rate of ADP release explains in part the approximately 4-fold faster unloaded velocity of the myosin-1C^0^ constructs. Our results are compatible with an additional contribution stemming from a 2-fold larger working stroke of Myo1C^0^-ΔTH1 than Myo1C^35^-ΔTH1, as predicted by the previously established structural models of the NTE^16^ and NTE^35^ peptides (22). According to these models, the NTEs form compact structural domains similar to the SH3-like domains of class II, V, and VI myosins, which are positioned near residues in the cleft between the motor domain and the calmodulin-binding region. In the case of NTE^35^, the model predicts the formation of a salt bridge between Arg21 and Glu469 in the relay loop (22). The presence of small independent NTE subdomains can sterically restrict the rotation of the lever arm and affect ADP release kinetics via allosteric pathways and thus account for the observed differences in motor activity (40).

## Experimental procedures

### Reagents

All chemicals and reagents were of the highest purity commercially available. *N*-(1-Pyrene)iodoacetamide was purchased from TFS (ThermoFisher Scientific, Waltham, MA, USA). Hepes, EGTA, potato apyrase (grade VII), and phalloidin were purchased from Sigma-Aldrich.

### Cloning, expression, and protein purification

Tail-truncated constructs Myo1C^35^-ΔTH1 and Myo1C^0^-ΔTH1 were coproduced with calmodulin in the baculovirus/*Sf9* insect cell system. The DNA sequences encoding the truncated myosin-1C isoforms with C-terminal octa-histidine tag were cloned into a pFastBac Dual vector (Invitrogen, Carlsbad, California, USA) under the control of the polyhedrin promoter with human calmodulin (CALM1; IMAGE ID 2821489) under the control of the p10. The truncated constructs were purified using immobilized metal-ion affinity chromatography on a Ni^2+^-NTA matrix and gel filtration on a Superdex 200 10/300 column (GE Healthcare Europe GmbH, Freiburg, Germany). Human full-length constructs Myo1C^35^-FL, Myo1C^16^-FL, and Myo1C^0^-FL were produced and purified in HEK293SF-3F6 cells as previously described (22). Calmodulin was produced tag-free in *E. coli* Rosetta pLySs (DE3) (Merck KGaA, Darmstadt, Germany) using vector pET-3a (Merck KGaA, Darmstadt, Germany) and purified using heat precipitation and a Phenyl Sepharose High Performance column (GE Healthcare Europe GmbH, Freiburg, Germany) as previously described (41). Purification of α-actin from chicken pectoralis major muscle was performed as previously described (42). Hexa-histidine–tagged human α-actinin 2 was produced in *E. coli* Rosetta pLySs (DE3) using vector pET-23a and purified on a Ni^2+^-NTA matrix (43). Proteins were used directly or flash-frozen with sucrose in liquid nitrogen and stored at −80 °C. Protein concentrations were determined by recording absorbance spectra of the region from 240 to 400 nm with a UV-2600 spectrophotometer (Shimadzu Deutschland GmbH, Duisburg, Germany). The molar extinction coefficient at 280 nm was calculated from the amino acid composition.

### Kinetic measurements

Steady-state ATPase assays were performed at 20° and 37 °C with the NADH-coupled assay in a buffer containing 25 mM imidazole (pH7.5), 25 mM KCl, 0.5 mM ATP, and 4 mM MgCl_2_ as described previously (44).

Transient kinetic experiments were performed at 20 °C in a buffer containing 25 mM Hepes, pH 7.5, 5 mM MgCl_2_, 0.5 mM DTT, and 50 mM KCl using either a HiTech Scientific SF-61 DX or a HiTech SF-61 SX stopped-flow system (TgK Scientific Ltd, Bradford-on-Avon, UK). All concentrations are given as final concentrations after rapid mixing. We used pyrene-labeled actin to track the actin association and dissociation of myosin-1C isoforms and how this is affected by ATP and ADP. Pyrene-labeled actin fluorescence was excited at 365 nm, and emission was monitored after passage through a KV-389 cutoff filter (Schott AG, Mainz, Germany). Intrinsic tryptophan fluorescence was excited at 295 nm, and emission was monitored after passage through a KV-320 cutoff filter (Schott AG, Mainz, Germany).

ATP-induced dissociation of myosin-1C from actin was determined by adding 0.03 to 10 mM ATP to 0.13 μM acto·Myo1C and monitoring the increase of the fluorescence signal. The ensuing change of the fluorescence signal was interpreted as a two-step process, as previously described (19, 24, 45). The model proposes a slow phase corresponding to the isomerization between two nucleotide-free actin-bound states A·M (open active site) and A·M' (closed active site), where only A·M is capable of binding ATP (Fig. 3). The fast phase represents the ATP binding to acto·Myo1C with subsequent dissociation of myosin-1C from actin.

ATP binding and hydrolysis was determined by adding 0.02 to 4 mM ATP to Myo1C-1IQ constructs and monitoring the resulting increase in the intrinsic tryptophan fluorescence signal (19).

Binding kinetics of myosin-1C to F-actin in the absence of nucleotides were determined by adding 0.25 to 3.00 μM pyrene-labeled actin to myosin-1C and monitoring the ensuing change in the amplitude of the fluorescence signal. Dissociation kinetics for the myosin-1C constructs from F-actin in the absence of ATP were determined by a chase experiment, where pyrene-labeled actin bound to myosin-1C was displaced by the addition of a large excess of unlabeled F-actin (46).

The apparent ADP affinity of the acto·Myo1C-ΔTH1 complexes can be assessed by ADP inhibition of the ATP-induced dissociation of the complexes (24, 25, 28). ADP binding and release was determined by adding 0.05 to 25 μM ADP to 0.13 μM acto·Myo1C and monitoring the increase of the fluorescence signal. In the presence of ADP, the *k*_obs_ of the slow phase is reduced with a hyperbolic dependence. At saturating concentrations of ADP, *k*_obs_ corresponds to the rate constant of ADP release (*k*_+5_). The affinity of A·M for ADP (*K*_5_) was determined by fitting the fraction of the slow amplitude according to A_slow_/A_total_ = [ADP]/(*K*_5_ + [ADP]) (28). The second-order rate constant of ADP binding (*k*_-5_) was calculated using the relationship *k*_-5_ = *k*_+5_/*K*_5_.

Phosphate release kinetics from acto·Myo1C were monitored using the HiTech Scientific SF-61 DX stopped-flow system with double mixing as previously described using N-[2-(1-maleimidyl)ethyl]-7-(diethylamino) coumarin-3-carboxamide–labelled phosphate-binding protein (MDCC-PBP) (47, 48). Myosin-1 C constructs and ATP were initially mixed and incubated for 6 s to allow ATP binding and hydrolysis to occur. This was followed by mixing with a 10-fold excess of actin (5 μM) to trigger phosphate release. Due to instrumental limitations related to the high viscosity of saturating actin concentrations, we determined an observed rate constant for the release of P_i_ by measuring the progress of the reaction in the presence of 5 μM actin. To determine the rate constant for P_i_ release (*k*_+4_), we performed kinetic simulations and global fitting.

Kinetic Studio software (TgK Scientific Ltd, Bradford on Avon, UK) was used for initial data inspection and analysis of transient kinetic data. Detailed data analysis was performed with Origin Pro 9.55 (OriginLab Corporation, Northampton, MA, USA) graphing and data analysis software. Each data point corresponds to the average of 3 to 5 single measurements. Goodness-of-fit criteria were evaluated using the coefficient of determination *R*^2^ and *χ*^2^ tests as implemented in Origin Pro 9.55. KinTek Explorer was used for global fitting with numerical integration (49). To constrain the simulation, we used the experimental data of the parameters 1/*K*_1_, *k*_+2_, *k*_+3_ + *k*_-3_, *k*_+5_, and *K*_α_ from transient kinetic measurements and *k*_cat_/*K*_app.actin_ from steady-state kinetic experiments. Errors of experimental data were included to the fit as statistical weighting parameters. To avoid local minima of the fit, we performed global fitting of the data several times with different initial rate constants.

The duty ratio corresponds to the fraction of time that myosin spends in strong binding states attached to F-actin during the ATPase cycle (Fig. 3). The rate of P_i_-release (*k*_+4_) is gating the transition from weak to strong F-actin–bound states, whereas the rate of ADP release (*k*_+5_) is gating the opposite transition. Accordingly, the duty ratio of myosin-1C is approximately equal to *k*_+4_/(*k*_+4_ + *k*_+5_) (36).

### *In vitro* motility assays

Unloaded *in vitro* motility assays were performed as described previously (50, 51) with some modification. Octa-histidine–tagged constructs Myo1C^0^-ΔTH1 and Myo1C^35^-ΔTH1 were immobilized on the glass surface using mouse monoclonal antibody QIAexpress Penta·His (Qiagen, Hilden, Germany). The surface density of the myosin-1C constructs were optimized by incubation with different antibody concentrations and by using a fixed antibody concentration by adding 10 μl of a 0.05 mg ml^−1^ antibody solution into the flow cell followed by incubation for 5 min at 18 °C, blocking with bovine serum albumin, and incubation for 5 min 18 °C with varying concentrations of myosin-1C constructs. Assays were performed using motor densities in the range from 180 to 5400 motors μm^-2^. In addition, we performed assays with 3600 motors μm^-2^ comparing the motile behavior of construct Myo1C^0^-ΔTH1 and Myo1C^35^-ΔTH1 in the presence and absence of peptides NTE^16^, NTE^35^, or NTE^35R21G^. To promote the formation of stable complexes, we added saturating concentrations of 50 μM NTE peptide to all assay solutions (22). All solutions were made up with assay buffer (20 mM imidazole pH 7.5, 50 mM KCl, 5 mM MgCl_2_, 2 mM EGTA). F-Actin was labelled with phalloidin-tetramethyl rhodamine B isothiocyanate (Merck KGaA, Darmstadt, Germany) overnight at 4 °C. The *in vitro* motility assay was started by the addition of 4 mM Mg^2+^-ATP in assay buffer containing oxygen scavengers and antibleach reagents (52). Actin sliding motility was measured at 37 °C using an Olympus IX70 fluorescence microscope equipped with a 60×/1.49 NA PlanApo objective and an Orca Flash 4.0 CMOS camera (Hamamatsu Photonics Deutschland GmbH, Herrsching, Germany). Tracking and analysis of filament movement were performed using the ImageJ plugin WrMTrck (Rasband, W.S., ImageJ, U. S. National Institutes of Health, Bethesda, Maryland, USA, https://imagej.nih.gov/ij/, 1997–2018) and Origin V9.55 (OriginLab Corporation, Northampton, MA, USA).

The unloaded sliding velocity of full-length constructs Myo1C^0^-FL, Myo1C^16^-FL, and Myo1C^35^-FL was determined on fluid membranes composed of physiological concentrations of PtdIns(4,5)P_2_. Flow cells containing a supported lipid bilayer on a coverslip were prepared in the following manner. 1,2-Dioleoyl-sn-glycero-3-phosphocholine and PtdIns(4,5)P_2_ were mixed in a molar ratio of 50:1, dried under vacuum, and resuspended in lipid buffer (25 mM Hepes pH 7.5, 100 mM KCl, 0.5 mM MgCl_2_). Small liposomes were prepared with intense vortexing and tip sonification. The glass coverslips were soaked in Piranha solution (3 parts sulphuric acid and 1 part 30 % hydrogen peroxide) and washed with deionized water. Liposomes containing PtdIns(4,5)P_2_ were incubated for 30 min in the flow cell with 2 mM CaCl_2_. Excess lipid mix was washed out with lipid buffer. One chamber volume of assay buffer containing the desired concentration of Myo1C-FL was added to the flow cell, followed by incubation for 5 min to allow attachment of Myo1C-FL constructs to the supported lipid bilayer. This was followed by the addition of one chamber volume containing tetramethylrhodamine-phalloidin–labelled actin filaments.

Frictional loading assays were performed with the Myo1C-ΔTH1 constructs bound to the surface-immobilized His antibody at a surface density of 3600 motors μm^-2^, except that α-actinin was used to generate a viscoelastic load on actin filaments (29). The relationship between stall force and concentration of α-actinin is given by Equation 1:

(1) $$F=\frac{\kappa }{k_{D}}\times \text{v}\times \text{ζ}\times L\times r\times \frac{k_{A}\times \text{χ}\times {\left[\alpha \right]}^{5/2}}{k_{A}\times \text{χ}\times {\left[\alpha \right]}^{3/2}+k_{D}}$$

where v is the filament sliding velocity, *k*_*A*_ and *k*_*D*_ are the second-order rate constants for acto·α-actinin attachment and detachment in the presence of ATP, respectively, *L* is the average length of a typical actin filament, *ζ* and *χ* are constants that define the surface concentration of α-actinin (29), *κ* is the elastic stiffness of the acto·Myo1C linkage with reported values from 0.2 to 0.5 pN nm^-1^ (45, 53), and *r* is the maximum distance for strong interactions between a surface-attached α-actinin molecule and an actin filament with a value of 61 nm (54, 55). The dependence of the sliding velocity on the stall forces was interpreted in terms of a sequential two-step model (Fig. 9), which was first proposed for *Mus musculus* myosin-1C^0^ (21, 28). The model defines the detachment of acto·myosin-1C as the result of a force-independent step and a force-dependent step, but the order of the steps cannot be distinguished by the model alone. The sum of the associated force-independent and the force-dependent detachment rate constants is equal to |*k*_a_ + *k*_b_|.

The relationship between the force-dependent sliding velocity v(F), both detachment rate constants and working stroke displacement *w*, is given by Equation 2:

(2) $$\text{v}\left(F\right)=k_{\text{det}}\left(F\right)\times w=\frac{w}{\frac{1}{k_{\text{i}}}+\frac{1}{k_{f_{0}}\times e^{\left(-\frac{F\times d}{k_{\text{B}}\times T}\right)}}}$$

where *d* is the distance parameter, *k*_f0_ is the detachment rate in the absence of external loads, *k*_B_ is Boltzmann’s constant, and *T* is the temperature. Power output *p* was calculated from the relationship between force produced by myosin motors and the velocity observed at this force (21).

## Data availability

All data are contained within the manuscript.

## Conflict of interest

The authors declare that they have no conflicts of interest with the contents of this article.
